# The New Offensive against Disease

**Published:** 1917-06-30

**Authors:** 


### THE NEW OFFENSIVE AGAINST DISEASE.

### King Edward VII.'s Hospital, Cardiff.

Adopting the phraseology of the battlefield, Colonel Bruce Vaughan, at a meeting of the management committee on June 13, sketched in detail the new "offensive" against disease" which is being undertaken by King Edward VII.'s Hospital, Cardiff. He spoke of great extensions in the work during the next few months or so. An orthopædic department for the treatment of civil and military patients for all kinds of injuries and deformities is to be opened by Dr. Fortescue Fox on July 5. Hydrology, massage, and electricity are included, and also radiant heat. Three types of remedial baths have been adopted: (1) sedative, pool baths; (2) tonic, douches; (3) local, whirlpool baths. The massage department, although not formally opened, is already attended by about eighty civilian patients. After many years of waiting, a convalescent home—thanks mainly to the enterprise and foresight of General H. H. Lee, chairman of the management committee—is to be opened next month in a beautiful situation at the seaside village of Sully. A beginning will be made with fifteen beds, reserved for women patients.

### The Nursing Department.

An improved scheme for the training of nurses is to be put in operation. Anthony House, Cardiff, is to be used as a preliminary training school for a maximum number of twelve probationer nurses, the opinion of Miss Luckes, matron of the London Hospital, being that the premises are excellently adapted for this purpose. An experienced teacher is to be appointed as sister-in-charge of this new development. The hospital proposes to alter its conditions of service, and will ask all candidates for training to enter into an agreement to serve the institution for five years—*i.e.*, three years' training in the hospital and two years on private nursing or on the general staff, as the matron may require. Another important alteration is the institution of a private nursing department, this being a return to the old order of things. The decision to provide every nurse who has given eighteen years' service with a pension of £60 per annum on retiring at the age of forty-five, without the nurse having to set aside any of her salary to secure it, is a most important step. Gold, silver, and bronze medals for efficiency in nursing have been given by Sir William James Thomas, and will be presented to the successful nurses shortly by Lady Thomas, formerly the assistant matron at the hospital.

### Child Welfare.

In respect to the venereal diseases campaign, additional work required for the wards and out-patient department has been carried out, and work has begun with about forty attendances of men, women, and children. Instruction given by Captain Owen Le Rhys in the technique of blood examination and modern methods of treatment is taken full advantage of by doctors. Big extensions in what may be termed the "child welfare" department are projected. These will soon be opened. Through the generosity of the Cory family, a ward of fourteen cots in memory of the late Mr. Richard Cory, and later a ward to accommodate twenty children, the gift of Mr. Charles Radcliffe, have been provided. This will make a total provision of one hundred children's beds. Then there are the splendid maternity wards now approaching completion; they have been provided by Mrs. John Nixon and others, and the plans for the Sir Edward Nicholl maternity hospital and child welfare department of seventy-five beds have been prepared. After unfolding this magnificent programme, Colonel Bruce Vaughan spoke of the hospital management's determination to do its part in placing Cardiff in its rightful position as the capital of Wales by the completion at the earliest practicable moment of the medical school and the adequate endowment of research. Another gift of 1,000 guineas to the hospital is announced, from Mr. C. A. G. Pullen, of Llanishen, in memory of his daughter; one of £50 from Mr. W. A. Boughton in memory of his late wife and as a thank-offering for the safety of his son, who had been reported missing at the Front; and another donation of £50 from Miss Dresser, of Lyndhurst, Hampshire.

